# Current landscape of T-cell engagers in early-phase clinical development in solid cancers

**DOI:** 10.3389/fimmu.2025.1665838

**Published:** 2025-10-06

**Authors:** Andrea Spinazzola, Giovanni Maria Iannantuono, James L. Gulley, Elena Giudice, Marco Filetti, Stefano Sganga, Francesca Lo Bianco, Charalampos S. Floudas, Gennaro Daniele

**Affiliations:** ^1^ Phase 1 Unit, Fondazione Policlinico Universitario A. Gemelli, Istituto di ricovero e cura a carattere scientifico (IRCCS), Rome, Italy; ^2^ Center for Immuno-Oncology, Center for Cancer Research, National Cancer Institute, National Institutes of Health, Bethesda, MD, United States; ^3^ Division of Gynecologic Oncology, Humanitas San Pio X, Milan, Italy

**Keywords:** solid tumors, T-cell engager, immunotherapy, early-phase trials, T lymphocytes

## Abstract

T-cell engagers (TCEs) are an emerging class of immunotherapeutic agents designed to harness the immune system to target and eliminate cancer cells. These molecules bridge T lymphocytes with tumor cells, generating an immunologic synapse that leads to potent immune-mediated tumor destruction. Although the clinical activity of TCEs in patients with solid tumors remains insufficient, recent technological advancements have led to the development of several candidates in early-phase clinical trials, with some showing encouraging signs of efficacy. This review examines the current landscape of TCEs in early clinical development for the treatment of solid tumors, describing their mechanism, clinical progress, efficacy, and challenges.

## 1 Introduction

Immunotherapies have revolutionized the approach to cancer treatment, with numerous strategies pursued over the last two decades. The core concept of immunotherapies is harnessing, through various means, the immune system of the host to eradicate the tumor. To date, the primary focus of cancer immunotherapy has been on T lymphocytes, which belong to the adaptive immune system and possess direct cytotoxicity against tumor cells (1–3).

A critical aspect of an efficient T cell-mediated response against cancer is the presence of tumor-associated antigens (TAAs) that T lymphocytes can recognize and react to. These typically include neoantigens, which are newly formed antigens that derive from mutations in the DNA. Other relevant types of TAAs encompass proteins whose expression is physiologically restricted to fetal development (known as oncofetal antigens), proteins that in the adult are expressed almost exclusively in the testes (known as cancer-testis antigens: CTAs), and proteins that are physiologically expressed by adult tissues but overexpressed by tumor cells (4, 5).

The most successful applications of immunotherapies involve monoclonal antibodies that block immunomodulatory molecules known as immune checkpoints, as well as chimeric antigen receptor-T (CAR-T) cells, which consist of engineered autologous T cells expressing a synthetic receptor against a TAA. While CAR-T cells offer a targeted therapeutic approach against cancer, immune checkpoint inhibitors (ICIs) work in part by inducing a robust generalized activation of T cells. Additional strategies that have been comprehensively explored, with mixed results, include vaccines targeting TAAs and the use of tumor-infiltrating lymphocytes (TILs) (1). Although immunotherapies have proven highly effective in prolonging the survival of patients with cancer, this benefit is generally restricted to only a subset of patients and to specific cancer types, and the extent of survival gain varies depending on the indication.

**Figure 1:**
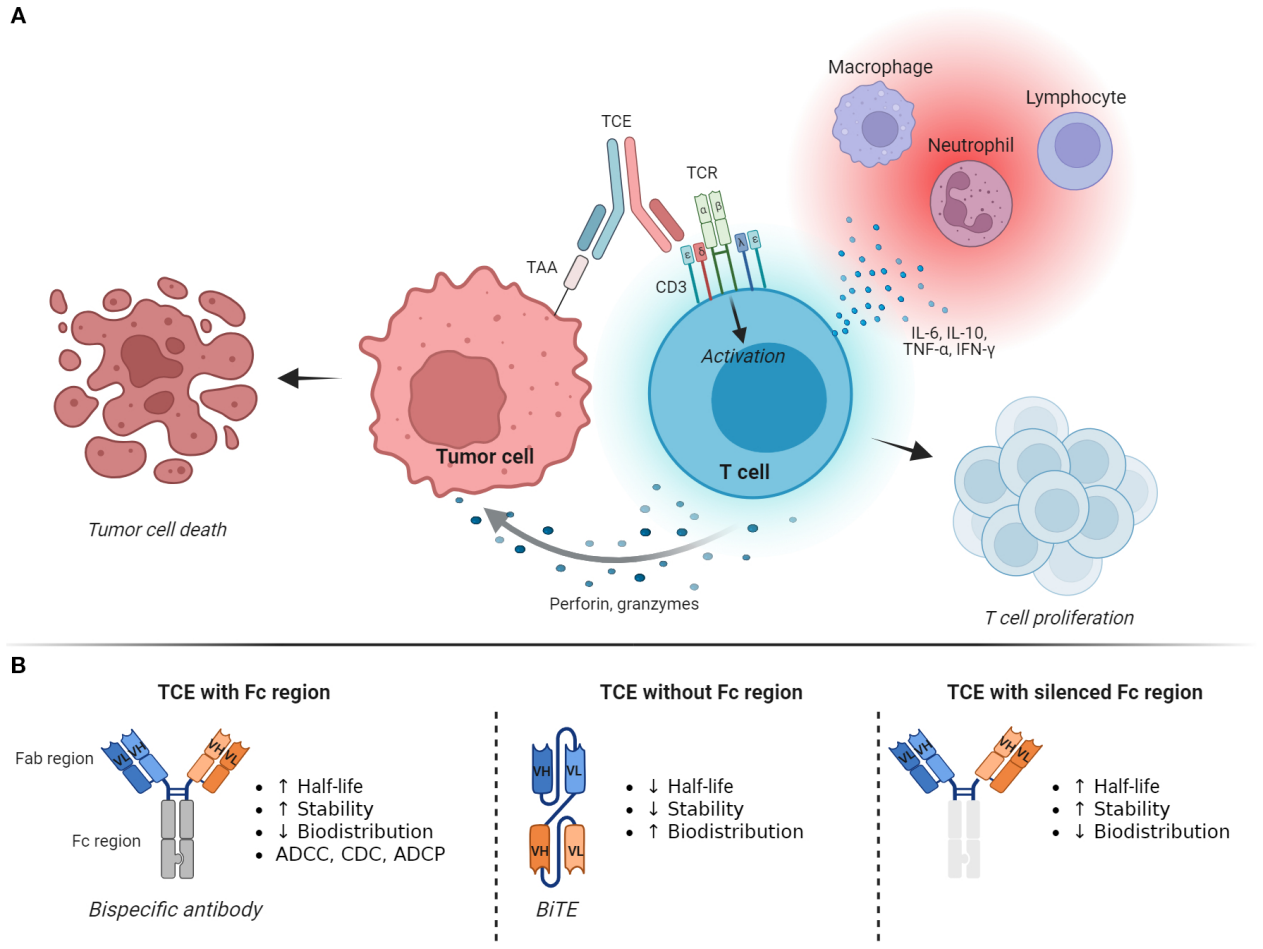
Mechanism of action and structure of T-cell engagers. **(A)** T-cell engagers (TCEs) generate an immunological synapse between tumor cells and T lymphocytes by binding a tumor-associated antigen (TAA) and the CD3ϵ subunit of the T-cell receptor (TCR) complex. This leads to T-cell activation, proliferation, and killing of the tumor cell through the release of perforin and granzymes. Activated T cells also produce and secrete various cytokines that induce inflammation and recruitment of other immune cells. **(B)** The basic structure of a TCE is represented by a bispecific antibody, which can be designed as an IgG-like full-length antibody that includes the Fc domain, as a fragment-based subtype that lacks the Fc region, or as a molecule with a silenced/effectorless Fc region. These changes confer different functional characteristics to the TCE, affecting its half-life, biodistribution, toxicity profile, and potentially its antitumor activity. Image created with Biorender.com. ADCC, antibody-dependent cell-mediated cytotoxicity; ADCP, antibody-dependent cell-mediated phagocytosis; BiTE, bispecific T-cell engager; CD3, cluster of differentiation 3; CDC, complement-dependent cytotoxicity; Fab region, fragment antigen-binding region; Fc region, fragment crystallizable region; IFN-γ, interferon-gamma; IL-6, interleukin-6; IL-10, interleukin-10; TAA, tumor-associated antigens; TCE, T-cell engager; TCR, T-cell receptor complex; TNF-α, tumor necrosis factor-alpha.

T-cell engagers (TCEs) function by simultaneously binding one or more TAAs and the CD3ϵ subunit of the T-cell receptor (TCR) complex expressed by T lymphocytes. This engagement redirects T cells to cancer cells, with TCEs bridging an effective immune synapse independently of the epitope specificity of the lymphocyte. As a result, T cells are activated and promoted to proliferate, produce cytokines, and selectively kill tumor cells through the release of perforin, which induces the formation of pores in the plasma membrane, along with granzymes, a family of serine proteases that cleave various intracellular proteins to induce cell death through apoptosis (6) (**Figure 1A**). A key feature of this process is that, unlike the antitumor immune response that occurs naturally or after treatment with ICIs, cancer vaccines, or TILs, it does not depend on antigen recognition on major histocompatibility complex (MHC) molecules. This enables T cells to attack cancer cells that do not express MHC molecules or whose TAAs are not efficiently mounted on MHC molecules, both of which are mechanisms that contribute to tumor immune evasion and resistance to immunotherapies (7, 8). Additionally, in contrast to ICIs and cancer vaccines, TCEs induce T cell-mediated lysis of tumor cells without requiring activation by antigen-presenting cells or the engagement of costimulatory molecules, and their activity does not depend on the epitope specificity of the TCR. Accordingly, TCEs present several advantages over traditional immunotherapies, offering a specific, effective, and immediate approach to enhance antitumor immunity that bypasses different steps of the complex process of T-cell activation against cancer cells.

The field of TCEs is rapidly advancing, with numerous new molecules entering clinical trials. However, in solid tumors, the development of many promising agents of this class is often discontinued due to the lack of meaningful activity, leading to a situation where most TCEs do not proceed to the late stages of clinical development (9). Accordingly, we provide an updated description of the current landscape of TCEs undergoing early-phase clinical evaluation in solid tumors, with a focus on efficacy data. We identified relevant phase 1 and 2 clinical trials on ClinicalTrials.gov and PubMed. Clinical data were obtained from published articles, abstract presentations at international conferences, and press releases from the sponsors, with a cutoff date of April 30, 2025. We prioritized TCEs that are currently undergoing clinical development or whose clinical trial results have been published within the last 5 years. For ease of narrative, we grouped the different TCEs based on the specific category of the TAAs they target.

## 2 TCEs under clinical evaluation in solid tumors

### 2.1 Structural and functional aspects

The basic structure of a TCE consists of a bispecific antibody (BsAb), an engineered artificial antibody that simultaneously binds two different antigens. As such, TCEs can be designed as IgG-like full-length antibodies that include the Fc domain or as fragment-based subtypes that lack an Fc region, such as bispecific T-cell engagers (BiTEs), nanobodies, and diabodies (6, 10, 11).

Functionally, these changes confer different characteristics that can be harnessed to optimize the design of the drug (**Figure 1B**). The presence of the Fc fragment confers a longer half-life, higher stability, and the potential for interactions with complement proteins and Fc receptors on innate immune cells. These interactions may enhance the antitumor effect of the drug through antibody-dependent cell-mediated cytotoxicity (ADCC), complement-dependent cytotoxicity (CDC), and antibody-dependent cell-mediated phagocytosis (ADCP). However, this design also carries disadvantages, such as its large size, which may limit tissue penetration and distribution, and possible adverse events (AEs) due to TAA-independent T-cell activation and ADCC and CDC toward the T cells (12, 13). On the other hand, TCEs that do not contain the Fc region exhibit better tissue penetration at the cost of lower stability and shorter plasma half-life. They also lack the potential antitumor effect related to ADCC, CDC, and ADCP. Molecules with silenced Fc fragments have been developed to abolish Fc-receptor binding and complement activation while retaining stability and extended half-life.

Additional strategies and innovative designs are being explored to improve patient safety and enhance antitumor activity. For instance, certain TCEs are administered as prodrugs that require proteolytic cleavage to unmask one or both of their binding domains (14). This technology leverages the high level of proteases typically found in the tumor microenvironment (TME), localizing the treatment in the tumor while remaining inactive in healthy tissues. Multispecific TCEs can bind to multiple TAAs or different epitopes of the same TAA, thereby increasing tumor selectivity and sensitivity. Alternatively, they can contain domains that engage ligands and receptors involved in T-cell activation, further stimulating antitumor immunity (15). Finally, TCEs may contain an additional binding domain for human serum albumin (HSA), which extends their half-life in the bloodstream (16).

The majority of available TCEs target TAAs that are localized on the cell membrane. This limits their applications and efficacy in solid tumors for two reasons. First, most TAAs are expressed as intracellular proteins, which can be exposed on the surface only as peptide fragments bound to MHC molecules. Secondly, many cell membrane-bound TAAs are also expressed by normal cells, which can result in on-target off-tumor toxicity (6, 17). To overcome these obstacles, various strategies have been pursued, most notably the development of molecules that combine a soluble TCR-targeting domain with an anti-CD3 effector domain, such as ImmTAC and TCER biologics. In contrast to classical TCEs, these agents redirect T cells toward tumor cells expressing a peptide fragment of the antigen presented by specific MHC class I molecules (18, 19).

**Figure 2:**
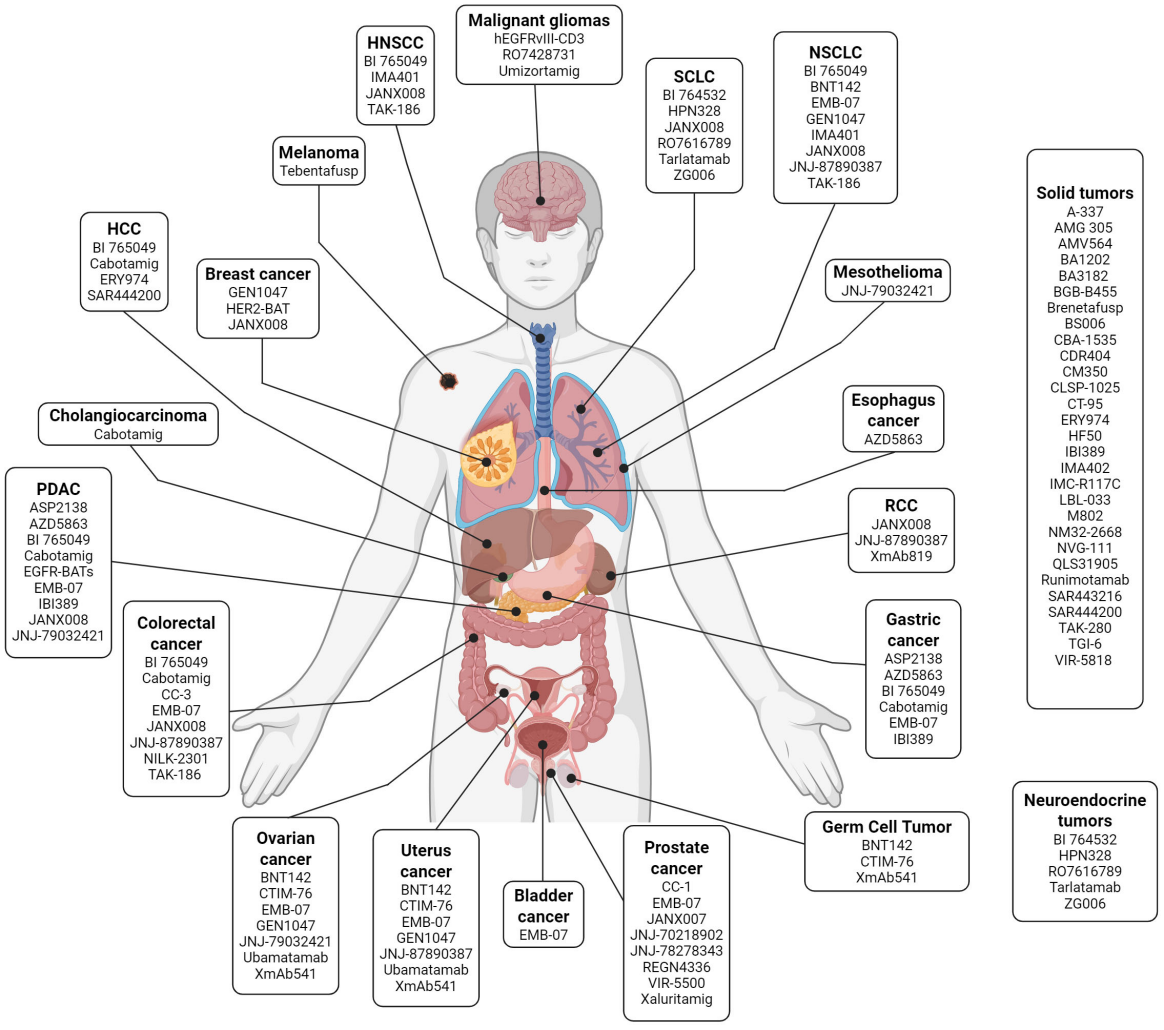
T-cell engagers in clinical development grouped by indication. List of selected T-cell engagers that are currently undergoing early-phase clinical development according to the tumor histology where they have been tested. Image created with Biorender.com. HNSCC, head and neck squamous cell carcinoma; HCC, hepatocellular carcinoma; NSCLC, non-small cell lung cancer; PDAC, pancreatic ductal adenocarcinoma; RCC, renal cell carcinoma; SCLC, small-cell lung cancer.

**Figure 3:**
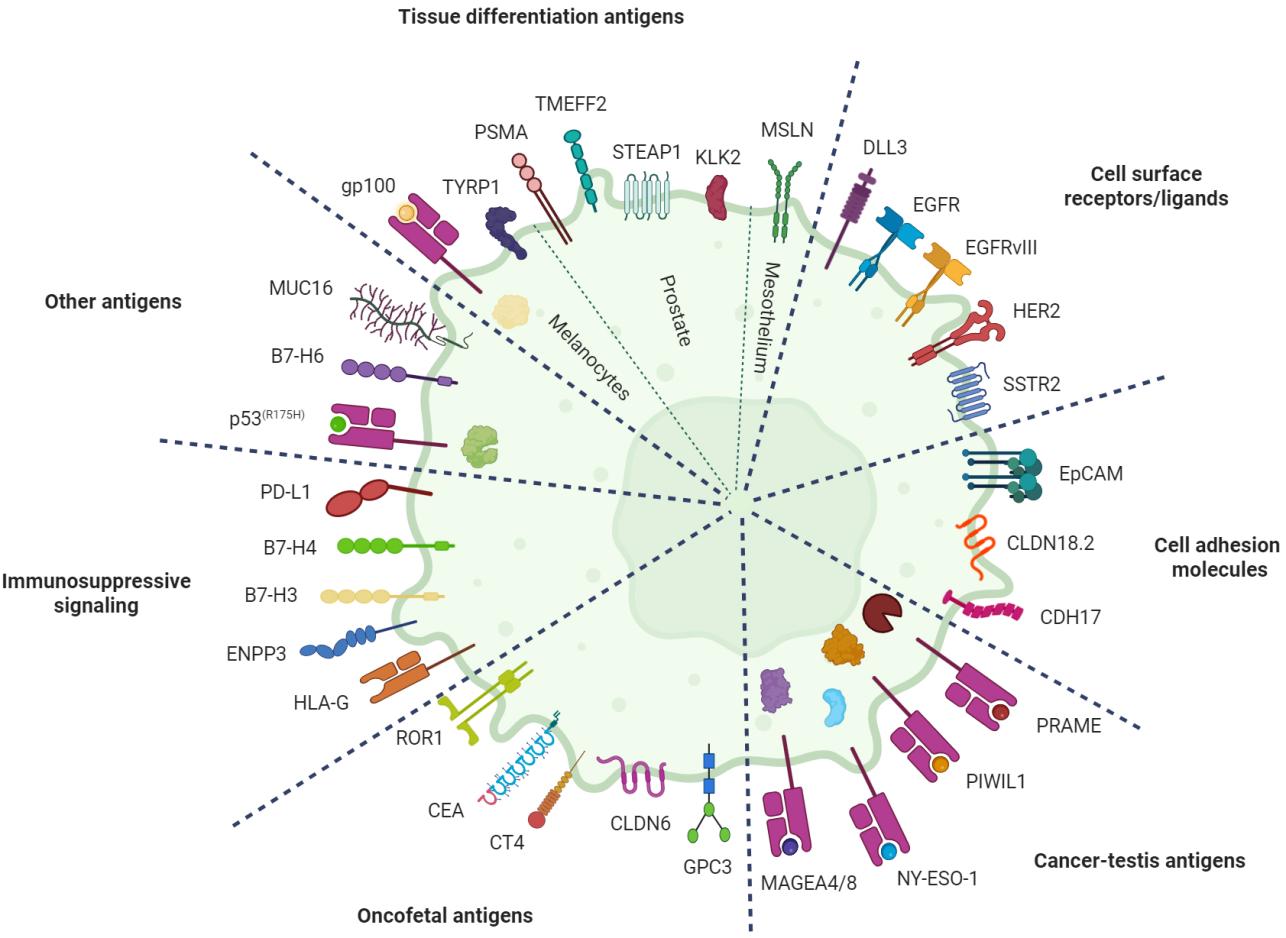
Targets for T-cell engagers in solid tumors. Illustration of the tumor-associated antigens (TAAs) that have been explored as targets for T-cell engagers in patients with solid tumors divided by category. Intracellular antigens are exposed on the plasma membrane as peptide fragments bound to major histocompatibility complex (MHC) class I molecules. Image created with Biorender.com. B7-H3, B7 homolog 3; B7-H4, B7 homolog 4; B7-H6, B7 homolog 6; CEA, carcinoembryonic antigen; CDH17, cadherin 17; CLDN18.2, claudin 18 isoform 2; CLDN6, claudin 6; CT4, cancer/testis antigen 4; DLL3, delta-like ligand 3; EGFR, epidermal growth factor receptor; EGFRvIII, epidermal growth factor receptor variant III; ENPP3, ectonucleotide pyrophosphatase/phosphodiesterase 3; EpCAM, epithelial cell adhesion molecule; GPC3, glypican 3; gp100, glycoprotein 100; HER2, human epidermal growth factor receptor 2; HLA-G, human leukocyte antigen G; KLK2, kallikrein 2; MAGEA4/8, melanoma-associated antigen A4/A8; MUC16, mucin 16; MSLN, mesothelin; NY-ESO-1, New York esophageal squamous cell carcinoma 1; PD-L1, programmed death-ligand 1; PIWIL1, P-element induced wimpy testis-like protein 1; PRAME, preferentially expressed antigen in melanoma; PSMA, prostate-specific membrane antigen; ROR1, receptor tyrosine kinase-like orphan receptor 1; SSTR2, somatostatin receptor 2; STEAP1, six-transmembrane epithelial antigen of the prostate 1; TMEFF2, transmembrane protein with EGF-like and two follistatin-like domains 2; TYRP1, tyrosinase-related protein 1.

**Table 1** provides an overview of the TCEs currently undergoing clinical development in solid tumors. **Figure 2** displays these agents based on their clinical indication, while **Figure 3** illustrates the TAAs that have been attempted as targets.

**Table 1:** T-cell engagers undergoing early clinical development in patients with solid tumors.

	TCE			Clinical trial				
	Name	Tumor antigen(s)	Other targets	Identifier	Phase	Indication	Other intervention(s)	Status
TISSUE DIFFERENTIATION ANTIGENS	Tebentafusp	gp100		NCT02535078	1	Advanced cutaneous melanoma (first-line/pretreated) in HLA-A*02:01+ patients	Durvalumab, tremelimumab	Completed
				NCT05315258	2	Cutaneous and uveal melanoma in molecular relapsed disease in HLA-A*02:01+ patients		Recruiting
	REGN4336	PSMA		NCT05125016	1/2	mCRPC (pretreated)	REGN5678, cemiplimab	Recruiting
	CC-1	PSMA		NCT05646550	1	Prostate cancer in BCR		Recruiting
				NCT04104607	1	mCRPC (pretreated)		Recruiting
	JANX007	PSMA	HSA	NCT05519449	1	mCRPC (pretreated)		Recruiting
	VIR-5500 (AMX-500)	PSMA		NCT05997615	1/2	mCRPC (pretreated)		Recruiting
	Xaluritamig (AMG 509)	STEAP1		NCT06613100	1	Localized prostate cancer (neoadjuvant)		Recruiting
				NCT06555796	1	Prostate cancer in BCR		Recruiting
				NCT04221542	1	mCRPC (pretreated)		Recruiting
	JNJ-70218902	TMEFF2		NCT04397276	1	mCRPC (pretreated)		Active
	JNJ-78278343	KLK2		NCT05818683	1	mCRPC (pretreated)	ARPI/docetaxel/cabazitaxel/cetrelimab	Recruiting
				NCT04898634	1	mCRPC (pretreated)		Recruiting
				NCT06095089	1	mCRPC (pretreated)	JNJ-87189401	Recruiting
	JNJ-79032421	MSLN		NCT06255665	1	Advanced: mesothelioma, ovarian cancer, PDAC - (pretreated)		Active
	CT-95	MSLN		NCT06756035	1	Advanced solid tumors (pretreated)		Recruiting
	AMG 305	MSLN, p-cadherin		NCT05800964	1	Advanced solid tumors (pretreated)		Recruiting
CELL SURFACE RECEPTORS/LIGANDS	Tarlatamab	DLL3		NCT05361395	1	Extensive-stage SCLC (first-line)	Atezolizumab/durvalumab/carboplatin, etoposide	Active
				NCT06830694	2	Transformed SCLC (first-line)	Atezolizumab, etoposide, carboplatin	Not yet recruiting
				NCT06814496	1/2	Advanced tumors with high prevalence of DLL3 (pretreated)	Radiotherapy	Not yet recruiting
				NCT04702737	1	Advanced NEPC (pretreated)		Completed
				NCT06816394	2	Advanced NEC - (pretreated)		Not yet recruiting
	BI 764532	DLL3		NCT04429087	1	Advanced SCLC, NET - (pretreated)		Recruiting
				NCT05882058	2	Advanced SCLC, NET - (pretreated)		Active
	HPN328 (MK-6070)	DLL3	HSA	NCT04471727	1/2	Advanced SCLC, NET, NEPC - (pretreated)	Atezolizumab/ifinatamab deruxtecan	Recruiting
				NCT06780137	1/2	Extensive-stage SCLC (pretreated)	Ifinatamab deruxtecan	Recruiting
	RO7616789 (RG6524)	DLL3	CD137	NCT05619744	1	Advanced SCLC, NEC - (pretreated)		Completed
	ZG006	DLL3		NCT05978284	1/2	Advanced SCLC, NEC - (pretreated)		Recruiting
				NCT06283719	1/2	Advanced SCLC, NEC		Recruiting
				NCT06440057	1/2	Advanced SCLC, NEC		Recruiting
				NCT06592638	1	SCLC (pretreated)		Recruiting
	JANX008	EGFR	HSA	NCT05783622	1	Advanced NSCLC, SCLC, HNSCC, CRC, PDAC, TNBC, RCC - (pretreated)		Recruiting
	TAK-186 (MVC-101)	EGFR		NCT04844073	1/2	Advanced: NSCLC, CRC, HNSCC - (pretreated)		Active
	EGFR-BATs	EGFR		NCT04137536	1	Advanced PDAC (pretreated)		Active
				NCT06479239	1/2	Advanced PDAC (pretreated)		Recruiting
	Umizortamig (GNC-039)	EGFRvIII	CD137, PD-L1	NCT04794972	1	HGG, glioblastoma, solid tumors - (pretreated)		Recruiting
	RO7428731	EGFRvIII		NCT05187624	1	HGG, glioblastoma (recurrent post-adjuvant therapy)		Active
	hEGFRvIII-CD3	EGFRvIII		NCT04903795	1	HGG, glioblastoma (recurrent post-adjuvant therapy)		Not yet recruiting
	SAR443216	HER2	CD28	NCT05013554	1	Advanced HER2+/mutated solid tumors (pretreated)		Terminated
	M802	HER2		NCT04501770	1	Advanced HER2+ solid tumors (pretreated)		N/A
	HF50	HER2		NCT06822998	1	Advanced HER2+/low solid tumors (pretreated)		Not yet recruiting
	VIR-5818 (AMX-818)	HER2		NCT05356741	1/2	Advanced HER2+ solid tumors (pretreated)	Pembrolizumab	Recruiting
	Runimotamab	HER2		NCT03448042	1	Advanced HER2+ solid tumors (pretreated)	Trastuzumab	Active
	HER2-BAT	HER2		NCT03272334	1/2	mBC HER2+ (pretreated)	Pembrolizumab	Active
CELL ADHESION MOLECULES	A-337	EpCAM		NCT06093698	1	Advanced solid tumors (pretreated)		Not yet recruiting
	BA3182	EpCAM		NCT05808634	1	Advanced adenocarcinomas (pretreated)		Recruiting
	M701	EpCAM		NCT06266091	2	Malignant ascites		Active
				NCT05543330	1/2	Malignant pleural effusion NSCLC		N/A
	ASP2138	CLDN18.2		NCT05365581	1	Advanced gastric cancer, PDAC (first-line, pretreated)	Chemotherapy (mFOLFIRINOX, mFOLFOX6, paclitaxel)/ramucirumab	N/A
	QLS31905	CLDN18.2		NCT05278832	1	Advanced solid tumors (pretreated)		N/A
	IBI389	CLDN18.2		NCT05164458	1	Advanced CLDN18.2+ solid tumors, especially GI (pretreated)	Sintilimab	Recruiting
	AZD5863	CLDN18.2		NCT06005493	1/2	Advanced gastric cancer, esophageal cancer, PDAC – (pretreated)		Recruiting
	Cabotamig (ARB202)	CDH17		NCT05411133	1	Advanced GI tumors (pretreated)	Atezolizumab	Recruiting
CANCER-TESTIS ANTIGENS	CDR404	MAGE-A4		NCT06402201	1	Advanced MAGE-A4+ solid tumors (pretreated) in HLA-A*02:01+ patients		Recruiting
	IMA401	MAGE-A4/8		NCT05359445	1	Advanced NSCLC, HNSCC, others – (pretreated) in HLA-A*02:01+ patients	Pembrolizumab	Recruiting
	IMA402	PRAME		NCT05958121	1/2	Advanced solid tumors (pretreated) in HLA-A*02:01+ patients		Recruiting
	Brenetafusp	PRAME		NCT04262466	1/2	Advanced solid tumors (pretreated) in HLA-A*02:01+ patients	Pembrolizumab/ chemotherapy/tabentafusp/bevacizumab/TKI	Recruiting
	IMC-R117C	PIWIL1		NCT06840119	1/2	Advanced solid tumors (pretreated) in HLA-A*02:01+ patients	Chemotherapy, antiangiogenic agents	Recruiting
ONCOFETAL ANTIGENS	ERY974	GPC3		NCT05022927	1	HCC (first-line)	Atezolizumab, bevacizumab	Active
				NCT02748837	1	Advanced GCP3+ solid tumors (pretreated)		Completed
	SAR444200	GPC3		NCT05450562	1/2	Advanced GCP3+ HCC and other solid tumors – (pretreated)	Atezolizumab	Active
	CM350	GPC3		NCT05263960	1/2	Advanced solid tumors (pretreated)		Recruiting
	NILK-2301	CEA		NCT06663839	1	Advanced CRC (pretreated)		Recruiting
	BA1202	CEA		NCT05909241	1	Advanced solid tumors (pretreated)		Recruiting
	GNC-035	ROR1	CD137, PD-L1	NCT05160545	1	Advanced breast cancer (pretreated)		Recruiting
	NM32-2668	ROR1	HSA	NCT06299163	1	Advanced solid tumors (pretreated)		Recruiting
	EMB-07	ROR1		NCT05607498	1	Advanced TNBC, NSCLC, ovarian cancer, uterus cancer, GI tumors, prostate cancer, bladder cancer – (pretreated)		Recruiting
	NVG-111	ROR1		NCT04763083	1	Advanced ROR1+ tumors (pretreated)		Recruiting
	XmAb541	CLDN6		NCT06276491	1	Advanced ovarian cancer, uterine cancer, testicular cancer – (pretreated)		Recruiting
	CTIM-76	CLDN6		NCT06515613	1	Advanced CLDN6+ gynecological tumors and testicular cancer – (pretreated with platinum)		Recruiting
	BGB-B455	CLDN6		NCT06803680	1	Advanced solid tumors (pretreated)		Recruiting
	BNT142	CLDN6		NCT05262530	1/2	Advanced CLDN6+ testicular cancer, ovarian cancer, endometrial cancer, non-squamous NSCLC – (pretreated)		Recruiting
	CBA-1535	5T4		jRCT2031210708	1	Advanced 5T4+ tumors (pretreated)	Pembrolizumab	Recruiting
IMMUNOSUPPRESSIVE SIGNALING	TAK-280	B7-H3		NCT05220098	1/2	Advanced solid tumors (pretreated)		Active
	CC-3	B7-H3		NCT05999396	1	Advanced CRC (pretreated)		Recruiting
	GEN1047	B7-H4		NCT05180474	1/2	Advanced breast cancer, ovarian cancer, endometrial cancer, squamous NSCLC – (pretreated)		Active
	BS006	PD-L1		NCT05938296	1	Advanced solid tumors (pretreated)		Recruiting
	XmAb819	ENPP3		NCT05433142	1	Advanced clear cell RCC (pretreated)		Recruiting
	JNJ-87890387	ENPP3		NCT06178614	1	Advanced RCC, endometrial cancer, CRC, non-squamous NSCLC – (pretreated)		Recruiting
	AMV564		CD33	NCT04128423	1	Advanced solid tumors (pretreated)	Pembrolizumab	Active
OTHER ANTIGENS	Ubamatamab (REGN4018)	MUC16		NCT06787612	2	Advanced platinum-resistant ovarian cancer	Bevacizumab/cemiplimab/fianlimab/pegylated liposomal doxorubicin	Not yet recruiting
				NCT03564340	1/2	Advanced ovarian cancer, endometrial cancer – (pretreated)	Cemiplimab	Recruiting
	LBL-033	MUC16		NCT05779163	1/2	Advanced solid tumors		Recruiting
	CLSP-1025	p53^(R175H)^		NCT06778863	1	Advanced TP53^R175H^+ tumors in HLA-A*02:01+ patients		Recruiting
	BI 765049	B7-H6		NCT04752215	1	Advanced GI tumors, NSCLC, HNSCC – (pretreated)	Ezabenlimab	Completed
				NCT06091930	1	Advanced GI tumors, NSCLC, HNSCC – (pretreated)	Ezabenlimab	Recruiting
				NCT06882746	1	Advanced GI tumors (pretreated)		Recruiting
	TGI-6	B7-H6		NCT06374173	1	Advanced B7-H6+ tumors (pretreated)		Recruiting

ARPI, androgen receptor pathway inhibitor; BCR, biochemical recurrence; CDH17, cadherin 17; CEA, carcinoembryonic antigen; CLDN6, claudin 6; CLDN18.2, claudin 18.2; CRC, colorectal cancer; DLL3, delta-like ligand 3; EGFR, epidermal growth factor receptor; EGFRvIII, epidermal growth factor receptor variant III; ENPP3, ectonucleotide pyrophosphatase/phosphodiesterase 3; EpCAM, epithelial cell adhesion molecule; GI, gastrointestinal; gp100, glycoprotein 100; GPC3, glypican-3; HCC, hepatocellular carcinoma; HER2, human epidermal growth factor receptor 2; HGG, high-grade glioma; HNSCC, head and neck squamous cell carcinoma; HSA, human serum albumin; KLK2, kallikrein-2; MAGE-A4, melanoma antigen gene A4; mBC, metastatic breast cancer; mCRPC, metastatic castration-resistant prostate cancer; MSLN, mesothelin; MUC16, mucin 16; N/A, not available; NEC, neuroendocrine carcinoma; NEPC, neuroendocrine prostate cancer; NET, neuroendocrine tumor; NSCLC, non–small cell lung cancer; PD-L1, programmed death-ligand 1; PDAC, pancreatic ductal adenocarcinoma; PIWIL1, Piwi-Like protein 1; PRAME, preferentially expressed antigen in melanoma; PSMA, prostate-specific membrane antigen; RCC, renal cell carcinoma; ROR1, receptor tyrosine kinase-like orphan receptor 1; SCLC, small cell lung cancer; STEAP1, six-transmembrane epithelial antigen of the prostate 1; TKI, tyrosine kinase inhibitors; TMEFF2, transmembrane protein with EGF-like and two follistatin-like domains 2; TNBC, triple-negative breast cancer.

### 2.2 TCEs targeting tissue differentiation antigens

The first milestone in the development of TCEs in solid tumors is approval by the U.S. Food and Drug Administration (FDA) of tebentafusp in January 2022 as first-line therapy for patients with advanced uveal melanoma (20). Tebentafusp is an ImmTAC that recognizes glycoprotein 100 (gp100) fragments mounted on MHC molecules encoded by the allele human leukocyte antigen (HLA)-A*02:01. As an essential component of the melanosome, the melanin-producing organelle in melanocytes, gp100 is also expressed in most cases of malignant tumors of melanocytic lineage (21). Efforts are ongoing to replicate the success obtained by tebentafusp in uveal melanoma in patients with cutaneous melanoma. A phase 1b trial assessing tebentafusp in combination with ICIs in 85 heavily pretreated patients with advanced cutaneous melanoma showed a response rate of 14% and a 1-year overall survival (OS) rate of 76% (22). Tebentafusp is also being evaluated in patients with uveal or cutaneous melanoma with molecular relapsed disease after removal of the primary tumor (NCT05315258).

Another potential melanocyte-specific target of TCEs is tyrosinase-related protein 1 (TYRP1), an additional constituent of the melanosome that is also expressed at low levels at the plasma membrane. Unfortunately, a phase 1 trial of the BsAb RO7293583 in patients with advanced TYRP1-positive cutaneous, uveal, or mucosal melanoma reported no objective response (23). This was attributed, at least in part, to the development of antidrug antibodies (ADAs) that resulted in reduced active drug exposure in a significant proportion of patients.

Prostate cancer is the second most frequent cancer and the fourth cause of cancer-related death in men worldwide (24). Besides the epidemiological relevance, different characteristics make prostate cancer an attractive target for immunotherapies. The prostate is a non-vital organ characterized by the expression of various proteins that are often conserved in prostate neoplasms and are not significantly expressed in other parts of the body. Among these, intense research has focused on prostate-specific membrane antigen (PSMA), a membrane enzyme that is overexpressed in 85%–100% of prostate cancers, especially in patients with metastatic disease (25). Results of various studies assessing TCEs targeting PSMA show limited efficacy of these agents as monotherapy in patients with metastatic castration-resistant prostate cancer (mCRPC). The objective response rate (ORR) ranged from 0% with the BiTe pasotuxizumab (26) or the Fc-silenced BsAb AMG 340 (27) to 7.4% with the half-life extended BiTE acapatamab (28). Only a minority of patients achieved a prostate-specific antigen (PSA) decrease of more than 50% compared with baseline (PSA50), a commonly used efficacy endpoint in clinical trials of mCRPC. Similar results were obtained with HPN424, which contains CD3-, PSMA-, and HSA-binding domains (29). Due to the lack of efficacy and a challenging safety profile, the clinical development of these four TCEs has been discontinued. It is important to highlight that prostate cancer is notoriously a tumor where other forms of immunotherapy have all shown limited efficacy (30, 31). Nonetheless, a new wave of PSMA-targeting TCEs has entered clinical development. Promising preliminary results show that almost all patients with mCRPC treated with the target dose of the BsAb CC-1 achieved some degree of PSA reduction (32). Preliminary data also show a PSA50 decline in 7 of 12 patients treated with the dual-masked TCE VIR-5500, a prodrug that is activated by tumor-associated proteases (33). The safety profile of this agent appears promising, with no patient presenting more than grade 2 cytokine release syndrome (CRS), a common AE associated with the use of TCEs and characterized by a systemic inflammatory response that can potentially lead to death due to respiratory failure, cardiac arrest, or multiorgan failure (34). JANX007 is a TRACTr biologic that contains PSMA- and CD3-binding domains, a peptide mask that prevents CD3 engagement on T cells, an albumin-binding domain attached to the mask, and a cleavable linker. To exert its therapeutic effect, after binding to PSMA-expressing tumor cells, JANX007 undergoes proteolytic cleavage to expose the CD3-engaging domain, while the albumin-binding domain is removed together with the mask to prevent the recirculation of the drug. Encouraging preliminary data from an ongoing phase 1 trial in patients with mCRPC show a PSA50 decline in all patients (n=16) treated with JANX007, with 75% of them maintaining the PSA decline after ≥12 weeks. The ORR was 50% among eight patients with measurable disease, and the drug was well tolerated (35).

Other targets for TCEs under investigation in prostate cancer include six-transmembrane epithelial antigen of prostate 1 (STEAP1), transmembrane protein with EGF-like and two follistatin-like domains 2 (TMEFF2), and kallikrein-2 (KLK2). Xaluritamig is a humanized antibody that contains two identical anti-STEAP1 binding domains, an anti-CD3 domain, and an effectorless Fc region. A phase 1 trial of xaluritamig monotherapy in patients with mCRPC reported that 24% of 67 evaluable patients and 41% of those in the target dose cohorts achieved a partial response (36). The PSA50 decline rates were 49% and 59%, respectively. At a median follow-up of 23.5 months, the median OS was 17.4 months (37). Although treatment-related adverse events (TRAEs) were observed in 97% of patients, they were serious in only 39% of patients. The most frequent were CRS (72% of all grades, 16% grade ≥3), fatigue (45%), and myalgia (34%) (36). Following these results, a phase 3 study (NCT06691984) has been launched to investigate xaluritamig versus cabazitaxel or an antiandrogen therapy in patients with pretreated mCRPC. JNJ-70218902 is a T cell-engaging BsAb targeting TMEFF2 that is being developed in patients with mCRPC. In an ongoing phase 1 study, JNJ-70218902 monotherapy led to a PSA50 decline in only 12.2% of patients, while the ORR among 33 patients with measurable disease was 15.2%. Toxicity was manageable, with 18.3% of patients experiencing TRAEs of grade ≥3, most frequently in the form of fatigue, lymphopenia, and asthenia (38).

### 2.3 TCEs targeting cell surface receptors/ligands

The second milestone in TCEs’ clinical advancement for solid tumors occurred in May 2024, following the FDA’s accelerated approval of tarlatamab for extensive-stage small-cell lung cancer (SCLC) pretreated with platinum-containing chemotherapy (39). Tarlatamab is a BiTE with an effectorless Fc fragment that targets delta-like ligand 3 (DLL3), a non-canonical inhibitory ligand of the Notch pathway. In normal cells, the expression of DLL3 is low and mainly confined to the Golgi apparatus and cytoplasmic vesicles. However, it is upregulated and relocates at the cell membrane in neuroendocrine neoplasms (40). Studies are now exploring tarlatamab, alone and in association with ICIs and chemotherapy in patients with treatment-naïve SCLC. Tarlatamab is also being studied in patients with extrapulmonary neuroendocrine tumors. For example, in patients with metastatic neuroendocrine prostate cancer (NEPC), preliminary data show that tarlatamab monotherapy resulted in an ORR of 10.5% among all patients and 22.2% in patients with DLL3-positive tumors (41). Additional TCEs targeting DLL3 are being investigated in patients with neuroendocrine tumors. Positive preliminary data from a phase 1/2 study of HPN328, which has a binding domain for HSA, show an ORR of 50% among patients with pretreated SCLC, 44% among patients with neuroendocrine carcinoma, and 36% among patients with NEPC. The drug was well tolerated, and although 59% of patients experienced CRS, it was grade ≥3 in only 3% of them (42). Promising efficacy and tolerability were also recently reported with the BsAb BI 764532 in a similar patient population (43).

The ErbB receptor family comprises four transmembrane receptors with tyrosine kinase activity, named ErbB 1 to 4, that bind to extracellular ligands and play essential roles in the initiation and progression of several types of solid tumors (44). The members of this family that have been more closely implicated in cancer physiopathology are epidermal growth factor receptor (EGFR; ErbB1) and human epidermal growth factor receptor 2 (HER2; ErbB2), with several targeted agents already approved by regulatory agencies. Numerous TCEs targeting EGFR, typically designed as prodrugs, are being tested in patients with advanced, treatment-refractory tumors of epithelial origin. For example, JANX008 is a TRACTr targeting EGFR with a similar structure as JANX007 (see above). Likewise, TAK-186 is a COBRA biologic targeting EGFR that also engages HSA and whose CD3 effector domain is unmasked by tumor proteases (45). CX-904 was a protease-activatable TCE whose clinical development has been discontinued. In a phase 1 study with preliminary data available, CX-904 demonstrated a very favorable safety profile and signs of activity, with an ORR of 33% and a disease control rate of 100% among six patients with pretreated pancreatic cancer (46).

Another strategy to engage T cells against EGFR-expressing tumors involves the use of EGFR-targeting bispecific antibody-armed activated T cells (BATs), consisting of autologous T cells that are expanded and conjugated *ex vivo* with a BsAb that binds to CD3 and EGFR. Their clinical development is currently focusing on patients with advanced pancreatic cancer. Results from an adaptive trial show clear signs of immune activation following the infusion of EGFR-BATs in patients with treatment-refractory pancreatic carcinoma. However, as monotherapy, the antitumor activity was modest, with no objective responses observed and two patients obtaining stable disease (47).

Mutations in EGFR that lead to a constitutively active receptor are common in certain tumor types. EGFRvIII is a tumor-specific EGFR variant that is detected in up to 19% of patients with glioblastoma (48), an aggressive type of brain tumor. A prematurely terminated phase 1 trial of the BiTE etevritamab showed that among eight patients with recurrent EGFRvIII-positive glioblastoma, only one achieved a partial response and two had a stable disease (49). However, approximately 50% of patients experienced serious AEs, most commonly in the form of headaches and depressed consciousness. Two additional TCEs, the BiTE hEGFRvIII-CD3 and the BsAb RO7428731, are being studied in patients with EGFRvIII-positive high-grade glioma or glioblastoma progressing after adjuvant radiotherapy and temozolomide. Moreover, a tetra-specific antibody construct named umizortamig, with binding domains for CD3, EGFRvIII, the immune checkpoint programmed death-ligand 1 (PD-L1), and the T-cell costimulatory receptor CD137, is being assessed in patients with relapsed or treatment-refractory malignant glioma as well as other solid tumors.

HER2 can act as an oncogene and drive tumor progression through either activating mutations or overexpression. The latter event is frequent in patients with certain types of cancer, such as breast cancer and gastric carcinoma, but can also be found in a minority of patients with other cancer histologies (50). Early attempts to target HER2-expressing tumors with TCEs have shown disappointing results. Two studies assessing the BsAbs ertumaxomab or GBR 1302 as single agents in patients with different types of HER2-positive tumors reported scarce to no objective responses (51, 52). Next-generation TCEs targeting HER2 that have entered clinical testing include the BsAbs M802 and runimotamab, the dual-masked prodrug VIR-5818, and the TRAFsome construct HF50. The latter consists of antibody fragments anchored onto liposomal surfaces with the aim of concentrating the drug in the TME due to enhanced permeability and retention effect compared with healthy tissues. Preliminary data from the cohorts of VIR-5818 monotherapy in a phase 1 study show that 50% of patients experienced some degree of tumor shrinkage, with two of six patients with treatment-refractory colorectal cancer achieving a partial response (33). This agent is well tolerated, with only a minority of patients presenting with grade 1–2 CRS and none with grade 3. SAR443216 is a tri-specific TCE that binds to HER2, CD3, and the T-cell costimulatory receptor CD28. Regrettably, a phase 1 trial showed a disappointing ORR of 0% among 40 patients with heavily pretreated HER2-positive or HER2-mutated tumors treated with SAR443216 (53).

HER2-directed BATs have also been developed. A study published some years ago reported scarce activity of HER2-BAT monotherapy in 23 patients with metastatic HER2-amplified breast cancer, with just one patient obtaining a partial response (54). A phase 1/2 trial is now assessing HER2-BATs in association with the ICI pembrolizumab in a similar patient population.

Finally, tidutamab was a T cell-engaging BsAb with a silent Fc fragment targeting somatostatin receptor 2 (SSTR2) that was tested in patients with advanced, well-differentiated neuroendocrine tumors (NETs). Although tidutamab was well tolerated and induced sustained T-cell activation and cytokine release, no objective response was observed (55). The development of this agent has been discontinued.

### 2.4 TCEs targeting cell adhesion molecules

Epithelial cell adhesion molecule (EpCAM) is a transmembrane glycoprotein mediating cell–cell adhesion in epithelial tissues. High levels of EpCAM expression are found in many carcinomas and have been associated with disease progression (56). Historically, the clinical application of TCEs targeting EpCAM has been hindered by the frequent occurrence of severe toxicities due to target abundance in normal tissues (57, 58). Nevertheless, their clinical development has continued. Preliminary data from a phase 2 randomized study (n=84) show that, after paracentesis, the intraperitoneal infusion of the BsAb M701 was effective in prolonging puncture-free survival compared with paracentesis alone among patients with malignant ascites due to epithelial cancer (59). A non-significant improvement in OS was observed, with 6-month survival rates of 32.3% for M701 and 12.6% for paracentesis alone. This agent was manageable, with 21.7% of patients experiencing serious TRAEs, primarily in the form of anemia, hypokalemia, and hyperglycemia (60). M701 is also being studied in patients with symptomatic malignant pleural effusion due to advanced non-small cell lung cancer (NSCLC), showing promising preliminary signs of activity and good tolerability (61). Another TCE targeting EpCAM is BA3182, a prodrug BsAb that is conditionally activated in the TME by the acidic pH. BA3182 is being tested as a single agent in patients with different types of adenocarcinomas.

Claudins (CLDNs) are a family of transmembrane proteins that are essential components of tight junctions. There are 26 members of this family in humans, with strong tissue- and cell-specific distribution. CLDN expression is dysregulated in various cancers and has been implicated in cancer-cell proliferation, invasion, migration, and metastasis (62). Various TCEs targeting CLDN18.2 are being studied, with a focus on patients with gastrointestinal tumors. Preliminary data show that approximately one-third of patients with treatment-refractory CLDN18.2-positive pancreatic adenocarcinoma or gastric cancer treated with the BsAb IBI389 obtained an objective response. Nevertheless, this was accompanied by significant toxicity, with more than half of patients experiencing grade ≥3 TRAEs (63, 64).

Cadherins (CDHs) are a family of numerous cell–cell adhesion molecules that can have both tumor-suppressive and tumor-promoting roles depending on the context and the specific member (65). Cabotamig, a TCE targeting CDH17, is currently under investigation in association with the ICI atezolizumab in patients with gastrointestinal tumors. The clinical development of PF-06671008, which targets p-cadherin, has been curbed due to a lack of efficacy (66).

### 2.5 TCEs targeting CTAs

Due to their limited distribution in adult cells and tissues outside the testes and their abundant expression in various tumor types, CTAs have long been considered an attractive target for anticancer therapy, particularly for immune-based interventions. Indeed, since T cells naturally recognize these antigens, efforts have been made to develop CTA-based vaccines. However, despite generating high frequencies of reactive T cells in the blood, no agent has shown consistent activity (67). Various TCEs targeting intracellular CTAs are under investigation in patients who carry the HLA-A*02:01 allele. Brenetafusp is an ImmTAC that engages preferentially expressed antigen in melanoma (PRAME). Preliminary results from an ongoing phase 1/2 study show that, in a cohort of 36 patients with cutaneous melanoma pretreated with ICIs, the ORR to brenetafusp monotherapy was 11%, with no responses observed in patients with PRAME-negative tumors (68). Similarly, in a cohort of 47 patients with platinum-resistant ovarian cancer, brenetafusp showed signs of activity both as monotherapy and in combination with chemotherapy (69). Brenetafusp appeared safe; the most frequently reported AE was grade 1–2 CRS in approximately half of patients in the monotherapy cohorts, predominantly during the first weeks of therapy. The clinical development of another ImmTAC named IMCnyeso which targets New York esophageal squamous cell carcinoma 1 (NY-ESO-1) has been discontinued after a phase 1 trial reported no objective response among 28 patients with solid tumors (70). IMA401 and IMA402 are two TCERs targeting melanoma antigen gene (MAGE)-A4/8-positive or PRAME-positive tumors, respectively. Encouraging early signs of activity accompanied by a manageable safety profile have been recently disclosed, and clinical trials are ongoing (71).

### 2.6 TCEs targeting oncofetal antigens

Glypican-3 (GPC3) is a membrane-bound heparan sulfate proteoglycan that is overexpressed in most cases of hepatocellular carcinoma (HCC) as well as in up to 45% of patients with squamous NSCLC and a minority of patients with other tumor types (72). A phase 1 trial of ERY974, a BsAb with a silenced Fc region, reported modest efficacy among 29 patients with GPC3-positive solid tumors, with only one patient achieving a partial response. The most frequent TRAEs were CRS, which was the dose-limiting toxicity (DLT), and pyrexia (73). SAR444200 is a GPC3-targeting TCE with two essential characteristics: structurally, it is a tandem of fragments from one domain of a heavy-chain antibody that is based on nanobody technology; functionally, it engages T cells via binding of TCRαβ subunit instead of CD3ϵ. Preliminary data from a phase 1/2 study show a ≥20% decrease in alpha-fetoprotein, a serum marker of HCC, in 2 of 18 patients with advanced HCC treated with SAR444200 (74).

Carcinoembryonic antigen (CEA) is a cell surface glycoprotein that belongs to the immunoglobulin gene superfamily and is often used as a marker in various types of epithelial tumors. Initial attempts to target CEA with TCEs have been negative, with cibisatamab and AMG 211 being discontinued after clinical studies reported scarce efficacy (75–77). New molecules include the BsAbs NILK-2301 and BA1202, which are being tested in patients with colorectal cancer and other solid tumors, respectively.

CLDN6 is a member of the CLDN family (see above) that is expressed especially in patients with gynecological tumors or germ cell tumors. While the development of BiTE AMG 794 has recently been halted, various T cell-engaging BsAbs are being developed. Moreover, BNT142 is a lipid nanoparticle-formulated RNA encoding a T cell-engaging BsAb targeting CLDN6. After intravenous administration, BNT142 accumulates in the liver, where the RNA is translated and the BsAb is self-assembled and then secreted into circulation (78). This formulation is predicted to improve the pharmacokinetic profile by prolonging the systemic availability of the TCE.

### 2.7 TCEs targeting immunosuppressive signaling

HLA-G is a non-classical MHC molecule with an immunosuppressive function whose physiological expression is restricted to the maternal–fetal interface and immune-privileged organs in the adult but can be aberrantly expressed in cancer (79). The TCE JNJ-78306358 was studied in heavily pretreated patients with tumors with a high prevalence of HLA-G expression. No objective responses were observed, and four patients experienced DLTs (80). The clinical development of JNJ-78306358 has been discontinued.

AMV564 is a tetravalent tandem diabody with two binding sites for CD33, a cell surface glycoprotein expressed by myeloid cells, and two for CD3ϵ (81). It has been tested in both myeloid neoplasms, where it directly induces the elimination of neoplastic cells, and in solid tumors, where it stimulates immune-mediated tumor clearance by depleting immunosuppressive myeloid-derived suppressor cells (MDSCs). Encouraging preliminary results from a phase 1 trial show that, in patients with treatment-refractory solid tumors, AMV564 induced clinical responses when administered both as monotherapy and in combination with pembrolizumab, including a complete response in a monotherapy-treated patient with ovarian cancer (82). Importantly, AMV564 was well tolerated, no DLT was observed, and the maximum tolerated dose (MTD) was not reached.

BS006 is an engineered recombinant type II herpes simplex oncolytic virus that encodes for a BsAb that redirects T cells toward PD-L1-positive tumor cells. Following intratumor injection, BS006-infected tumor cells are instructed to produce and secrete the TCE. Accordingly, BS006 is expected to induce a robust antitumor immune response that combines the inflammatory preconditioning of the TME due to the direct effect of the oncolytic virus plus the T cell-mediated response deriving from the action of the TCE and the inhibition of PD-L1. A phase 1 study is evaluating BS006 as a single agent in patients with advanced treatment-refractory solid tumors. Results are pending.

## 3 Overcoming the limits

**Figure 4:**
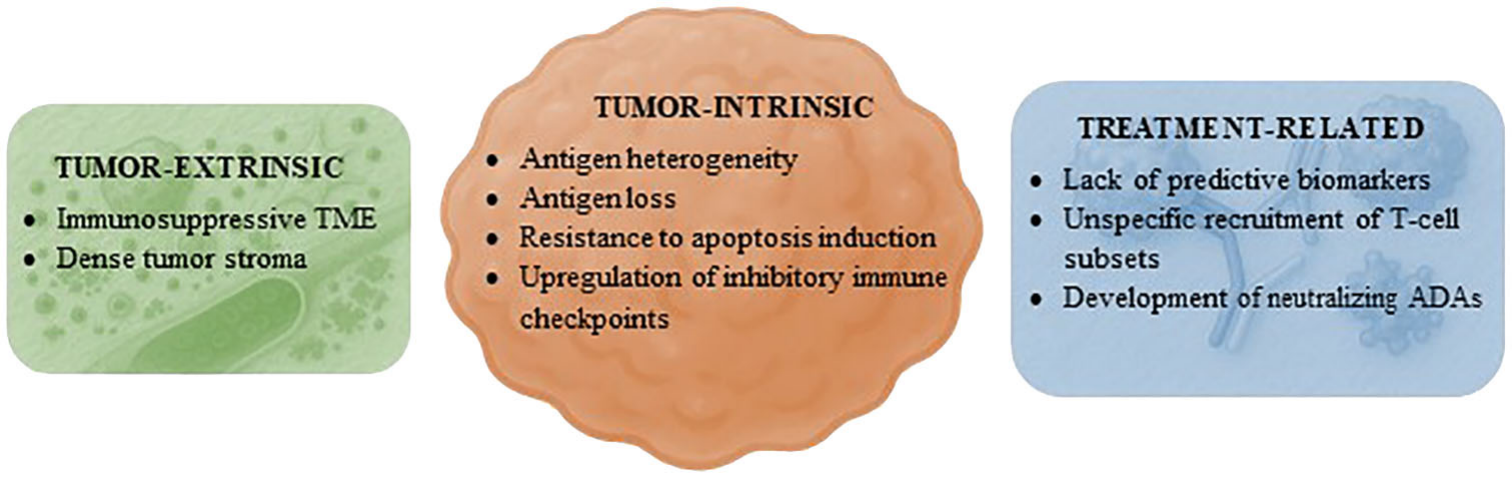
Mechanisms of resistance to T-cell engagers in solid tumors. Proposed factors hindering the efficacy of T-cell engagers in patients with solid tumors grouped by category. ADAs, antidrug antibodies; TME, tumor microenvironment.

Following the success in patients with B-cell hematologic malignancies and those with uveal melanoma or SCLC, the development of TCEs has generated intense interest and excitement. Nonetheless, most of the currently available TCEs provide only limited benefit in patients with solid tumors (**Table 2**). Like what has been observed in the field of CAR-T cells (83), TCEs have demonstrated significant success in treating B-cell neoplasms, with seven agents of this class already approved by regulatory agencies (**Supplementary Table S1**). However, their application in solid tumors presents additional challenges that have limited their efficacy (84) (**Figure 4**). These include the immunosuppressive TME, which may be devoid of T cells and/or contain immunosuppressive cells such as MDSCs and regulatory T cells (Tregs) that prevent the activation of T cells; the physical barrier posed by the tumor stroma, which contains a dense extracellular matrix and results in poor penetration of drugs; and antigen heterogeneity, which may lead to the emergence of clones of tumor cells that do not express the TAA targeted by the TCE. In addition, tumor-intrinsic resistance mechanisms such as antigen loss, resistance to apoptosis induction, and upregulation of inhibitory immune checkpoints may have contributed to this outcome (84).

**Table 2:** Clinical activity of T-cell engagers with final or interim results in advanced solid tumors.

	TCE			Clinical trial						
	Name	Design	Target(s)	Identifier	Phase	Indication	Status	N. patients	Clinical activity	Ref.
TISSUE DIFFERENTIATION ANTIGENS	Tebentafusp	ImmTAC	gp100	NCT02535078	1	Cutaneous melanoma in HLA-A*02:01+ patients	Completed	85	With durvalumab ± tremelimumab: ORR 14%, DoR 19.6 months, OS 18.7 months	(22)
	RO7293583	BsAb with two TYRP1-binding domains and one CD3-binding domain (2 + 1 format)	TYRP1	NCT04551352	1	Melanoma (TYRP1+)	Completed	20	Monotherapy: ORR 0%, OS NR/NA	(23)
	Pasotuxizumab	BiTE	PSMA	NCT01723475	1	mCRPC	Completed	47	Monotherapy: ORR 0%, PSA50 30%, OS NR/NA	(26)
	AMG 340	BsAb with a low-affinity CD3-binding domain	PSMA	NCT04740034	1	mCRPC	Completed	42	Monotherapy: ORR 0%, PSA50 9.7%, OS NR/NA	(27)
	Acapatamab	BiTE linked to an Fc fragment	PSMA	NCT03792841	1	mCRPC	Completed	133	Monotherapy (dose expansion cohort): ORR 7.4%, DoR 11.3 weeks, PSA50 30.4%, OS 10 months	(28)
	HPN424	TriTAC with HSA-binding domain	PSMA	NCT03577028	1/2	mCRPC	Terminated	80	Monotherapy: PSA50 4.7% (3 of 63), OS NR/NA	(29)
	CC-1	BsAb	PSMA	NCT04104607	1	mCRPC	Recruiting	28	Monotherapy: PSA decline in almost all patients treated with the target dose, OS NR/NA	(32)
	VIR-5500	Dual masked prodrug	PSMA	NCT05997615	1/2	mCRPC	Recruiting	12	Monotherapy: PSA50 58%, OS NR/NA	(33)
	JANX007	TRACTr with masked CD3-binding domain and an HSA-binding domain	PSMA	NCT05519449	1	mCRPC	Recruiting	16	Monotherapy: ORR 50%, PSA50 100%, DoR and OS NR/NA	(35)
	Xaluritamig	BsAb with two STEAP1-binding sites and an effectorless Fc fragment	STEAP1	NCT04221542	1	mCRPC	Recruiting	97	Monotherapy: ORR 24%, DoR 9.2 months, PSA50 49%, OS 17.4 months	(36, 37)
	JNJ-70218902	BsAb	TMEFF2	NCT04397276	1	mCRPC	Active	82	Monotherapy: ORR 15.2%, DoR 4.5-15.4 months, PSA50 12.2%, OS NR/NA	(38)
CELL SURFACE RECEPTORS/LIGANDS	Tarlatamab	BiTE linked to an effectorless Fc fragment	DLL3	NCT05060016	2	SCLC	Active	220	Monotherapy: ORR 40% (10mg)/32% (100mg), DoR ≥6 months 59%, OS at 9 months 68% (10mg)/66% (100mg)	(86)
				NCT04702737	1	NEPC	Completed	40	Monotherapy: ORR 10.5%/22.2% in DLL3+ tumors, OS NR/NA	(41)
	HPN328	TriTAC with HSA-binding domain	DLL3	NCT04471727	1/2	SCLC, NET, NEPC	Recruiting	66	Monotherapy: ORR 50% in SCLC/44% in NEC/36% in NEPC, OS NR/NA	(42)
	BI 764532	BsAb	DLL3	NCT04429087	1	SCLC, NET	Recruiting	90	Monotherapy: ORR 33% in SCLC/22% in NETs, OS NR/NA	(43)
	CX-904	Dual masked BsAb prodrug	EGFR	NCT04844073	1/2	Solid tumors	Active	35	Monotherapy: ORR 33% in PDAC (2 of 6), DoR and OS NR/NA	(46)
	EGFR-BATs	Engineered autologous T cells bound to a BsAb	EGFR	NCT02620865, NCT01420874	1/2	PDAC	Completed	8	Monotherapy: ORR 0%, OS NR/NA	(47)
	Etevritamab (AMG 596)	BiTE	EGFRvIII	NCT03296696	1	HGG, glioblastoma	Terminated	15	Monotherapy: ORR 12.5%, DoR and OS NR/NA	(49)
	Runimotamab	BsAb	HER2	NCT03448042	1	HER2+ tumors	Active	73	Monotherapy: ORR 30.4% in mBC, DoR and OS NR/NA	(87)
	VIR-5818	Dual masked prodrug	HER2	NCT05356741	1/2	HER2+ tumors	Recruiting	20	Monotherapy: ORR 33% in CRC (2 of 6), DoR and OS NR/NA	(33)
	Ertumaxomab	BsAb	HER2	NCT01569412	1/2	HER2+ tumors	Terminated	14	Monotherapy: ORR 7%, DoR and OS NR/NA	(51)
	GBR 1302	BsAb	HER2	NCT02829372	1	HER2+ tumors	Terminated	19	Monotherapy: ORR 0%, OS NR/NA	(52)
	SAR443216	Trispecific TCE including a domain targeting the T-cell costimulatory receptor CD28	HER2, CD28	NCT05013554	1	HER2+/mutated tumors	Terminated	40	Monotherapy: ORR 0%, OS NR/NA	(53)
	HER2Bi BAT	Engineered autologous T cells bound to a BsAb	HER2	NCT00027807	1	mBC (HER2+)	Completed	23	Monotherapy: ORR 4.5%, OS 36.2 months (57.4 months for HER2 3+/27.4 months for HER2 0–2+)	(54)
	Tidutamab	BsAb with a silent Fc fragment	SSTR2	NCT03411915	1	Well-differentiated NETs	Completed	41	Monotherapy: ORR 0%, OS NR/NA	(55)
CELL ADHESION MOLECULES	M701	BsAb	EpCAM	NCT06266091	2	Malignant ascites	Active	84	Puncture-free survival HR = 0.40, OS HR = 0.65	(59)
				NCT05543330	1/2	Malignant pleural effusion (NSCLC)	NA	24	Effusion volume decrease >50% in 61.5%, OS NR/NA	(61)
	QLS31905	BsAb	CLDN18.2	NCT05278832	1	Solid tumors	NA	52	Monotherapy: Monotherapy: ORR 11.1% (n. 27), OS NR/NA	(88)
	IBI389	BsAb	CLDN18.2	NCT05164458	1	CLDN18.2+ tumors (especially GI tumors)	Recruiting	114	Monotherapy: ORR (CLDN18.2 expression ≥10%) 30.4% in PDAC (7 of 23)/30.8% in gastric cancer (8 of 26), OS NR/NA	(63, 64)
	PF-06671008	Bispecific DART	CDH3	NCT02659631	1	Solid tumors	Terminated	27	Monotherapy: ORR 0%, OS NR/NA	(70)
CANCER-TESTIS ANTIGENS	Brenetafusp	ImmTAC	PRAME	NCT04262466	1/2	Solid tumors in HLA-A*02:01+ patients	Recruiting	N/A	Monotherapy: ORR 11% in PRAME+ melanoma (4 of 36)/0% in PRAME- melanoma (0 of 5)/7% in ovarian cancer (2 of 28). 6-month OS rate melanoma 95% in PRAME+/40% in PRAME-. With chemotherapy: ORR 25% in ovarian cancer (3 of 12)	(68, 69)
	IMCnyeso	ImmTAC	NY-ESO-1	NCT03515551	1	Solid tumors in HLA-A*02:01+ patients	Terminated	28	Monotherapy: ORR 0%, OS 3 months (3–10 μg)/12 months (30–300 μg)	(70)
	IMA402	TCER	PRAME	NCT05958121	1/2	Solid tumors in HLA-A*02:01+ patients	Recruiting	33	Monotherapy: ORR 9.5% (2 of 21 in PRAME+)/0% (0 of 7 in PRAME-), OS NR/NA	(71)
	IMA401	TCER	MAGE-A4/8	NCT05359445	1	NSCLC, HNSCC, others in HLA-A*02:01+ patients	Recruiting	35	Monotherapy: ORR 25% in 17 patients with relevant IMA401 doses and MAGEA4/8^high^ levels, OS NR/NA	(71)
ONCOFETAL ANTIGENS	ERY974	BsAb with a silent Fc fragment	GPC3	NCT02748837	1	GCP3+ tumors	Completed	29	Monotherapy: ORR 3.4%, OS NR/NA	(73)
	SAR444200	Nanobody	GPC3	NCT05450562	1/2	HCC, NSCLC (GCP3+)	Active	33	Monotherapy: AFP decrease ≥20% in 11.1% HCC (2 of 18), OS NR/NA	(74)
	Cibisatamab	BsAb with two CEA-binding domains and one CD3-binding domain (2 + 1 format) and a silent Fc fragment	CEA	NCT02324257	1	GI tumors	Completed	149	Monotherapy: ORR 4%, DoR 6.5 months, OS NR/NA	(75)
				NCT02650713	1	Solid tumors	Completed	228	Monotherapy and with Atezolizumab: ORR 6.6%, OS NR/NA	(75)
	AMG 211 (MEDI-565)	BiTE	CEA	NCT01284231	1	GI tumors	Completed	44	Monotherapy: ORR 0%, OS NR/NA	(76)
IMMUNOSUPPRESSIVE SIGNALING	JNJ-78306358	BsAb	HLA-G	NCT04991740	1	RCC, CRC, ovarian cancer	Completed	39	Monotherapy: ORR 0%, OS NR/NA	(80)
	AMV564	Bivalent bispecific (2 + 2 format)	CD33	NCT04128423	1	Solid tumors	NA	30	Monotherapy and with Pembrolizumab: ORR NR/NA (1 complete response in ovarian cancer), OS NR/NA	(82)
OTHER	Ubamatamab	BsAb	MUC16	NCT03564340	1/2	Ovarian cancer, endometrial cancer	Recruiting	109	Monotherapy (n. 42): ORR 14.3%, DoR 13.7 months in ovarian cancer, OS NR/NA With Cemiplimab (n. 22): ORR 18.2%, DoR 8.3 months in ovarian cancer, OS NR/NA	(89)

AFP, alpha-fetoprotein; BiTE, bispecific T cell engager; BsAb, bispecific antibody; CRC, colorectal cancer; DART, dual-affinity re-targeting; DoR, duration of response; GI, gastrointestinal; HR, hazard ratio; HCC, hepatocellular carcinoma; HGG, high-grade glioma; HNSCC, head and neck squamous cell carcinoma; ImmTAC, immune mobilizing monoclonal TCRs against cancer; mBC, metastatic breast cancer; mCRPC, metastatic castration-resistant prostate cancer; NA, not available; NEC, neuroendocrine carcinoma; NEPC, neuroendocrine prostate cancer; NETs, neuroendocrine tumors; NR, not reported; NSCLC, non–small cell lung cancer; ORR, objective response rate; OS, overall survival; PDAC, pancreatic ductal adenocarcinoma; PSA, prostate-specific antigen; PSA50, PSA decrease >50% compared with baseline; RCC, renal cell carcinoma; SCLC, small cell lung cancer; TCER, T-cell engaging receptors; TNBC, triple-negative breast cancer; TRACTr, tumor-activated T cell engager; TriTAC, tri-specific T-cell activating construct.

Factors inherent to the therapeutic agent or treatment strategy may also have hindered the successful application of TCEs in solid tumors. First, there is a lack of predictive biomarkers for efficacy beyond the expression of the TAA. Second, the administration of a TCE may recruit not only T lymphocytes with effector functions but also various other T-cell subsets. These may have immunosuppressive properties, such as naïve or exhausted T cells as well as Tregs, or they may increase treatment toxicity, including other CD4+ T-cell populations (85). Third, the development of neutralizing ADAs in response to the immunogenicity of TCEs is frequently reported in clinical studies (23, 26, 28, 36, 74, 75, 80). A strategy that has been implemented to attenuate this risk involves the pretreatment of patients with drugs that deplete B cells, such as anti-CD20 antibodies (75). Interestingly, numerous TCEs targeting DLL3 demonstrated consistent clinical activity in tumors of neuroendocrine lineage (39, 41–43, 86). In contrast, a TCE targeting SSTR2 failed to provide benefit in patients with NETs (55). This implies that, at least in some instances, accurately identifying the TAA that T cells should target in each tumor type may be crucial in determining the efficacy of these drugs, regardless of the chemical or pharmacological properties of the TCE.

The dose-finding studies included in this review suggest that the safety profile of current TCEs is not a prominent factor limiting their therapeutic potential. The clinical data made available during the last 5 years from completed or terminated studies, along with those from ongoing clinical trials, reveal that the MTD has been identified in only 19% of these studies, while it was not reached in 61% of them (**Supplementary Figure S1**). Indeed, various optimization strategies are generally applied to mitigate toxicity and decrease the incidence and severity of class-specific AEs such as CRS and neurotoxicity. Examples include step-up dosing to modulate the intensity of T-cell activation and cytokine release, subcutaneous administration to decrease the maximum concentration and provide a more gradual release of the drug into the bloodstream compared with the intravenous route, and prophylactic treatment with anti-interleukin-6 agents to prevent the cytokine storm (34, 90). An additional strategy involves the design of TCEs with a low-affinity binding site for CD3 to reduce cytokine release (91).

Continued innovation in TCE design, alongside a deeper understanding of its mechanisms of action and interaction with the TME, is essential for realizing its full therapeutic potential. For instance, combining TCEs with other therapeutic approaches such as ICIs, vaccines, or conventional chemotherapy might enhance overall efficacy and potentially prevent resistance. Numerous early-phase clinical trials are already testing TCEs in combination with diverse classes of antitumor medical therapies. The results of these cohorts will be highly informative in refining the therapeutic application of TCEs.

The identification of new TAAs that can be targeted could also facilitate the successful development of TCEs. Apart from TAAs that are common to different tumor histologies or shared by patients with the same tumor type, tailoring TCEs based on individual patient profiles could enhance therapeutic outcomes and minimize adverse effects. For example, after the molecular profiling of a tumor biopsy, a TCE could be designed to target one or more TAAs that are specific to each patient. This procedure could be repeated at the time of tumor progression to redirect treatment toward the emerging tumor-cell clones that drive resistance, thus providing a longitudinal, adaptive approach to cancer immunotherapy.

## 4 Conclusions

TCEs are a rapidly evolving class of therapeutic agents in oncology with the potential to significantly impact the treatment landscape for solid tumors. They represent a transformative, powerful, and targeted approach to cancer immunotherapy. Although expectations regarding TCEs have not yet been fulfilled in patients with solid tumors, encouraging results from a few contemporary studies bring hope for a change of direction. With a wealth of new TCEs being developed and tested in clinical trials, the next few years will be critical in providing a definitive verdict regarding their utility in solid tumors.

## Glossary

ADCC: antibody-dependent cell-mediated cytotoxicity
ADCP: antibody-dependent cell-mediated phagocytosis
AE: adverse event
BATs: bispecific antibody armed activated T cells
BiTE: bispecific T cell engager
BsAb: bispecific antibody
CAR-T: chimeric antigen receptor-T
CDC: complement-dependent cytotoxicity
CDH17: cadherin 17
CEA: carcinoembryonic antigen
CLDN6: claudin 6
CLDN18.2: claudin 18.2
CRS: cytokine release syndrome
DLL3: delta-like ligand 3
COBRA: COnditional bispecific redirected activation
DLT: dose-limiting toxicity
EGFR: epidermal growth factor receptor
EGFRvIII: epidermal growth factor receptor variant III
EpCAM: epithelial cell adhesion molecule
FDA: Food and Drug Administration
gp100: glycoprotein 100
GPC3: glypican-3
HCC: hepatocellular carcinoma
HER2: human epidermal growth factor receptor 2
HLA: human leukocyte antigen
HSA: human serum albumin
ICI: immune checkpoint inhibitor
ImmTAC: immune mobilizing monoclonal TCRs against cancer
KLK2: kallikrein-2
MAGE-A4: melanoma antigen gene A4
mCRPC: metastatic castration-resistant prostate cancer
MDSC: myeloid-derived suppressor cells
MHC: major histocompatibility complex
MTD: maximum tolerated dose
NEPC: neuroendocrine prostate cancer
NET: neuroendocrine tumor
NSCLC: non–small cell lung cancer
NY-ESO-1: New York esophageal squamous cell carcinoma 1
ORR: objective response rate
OS: overall survival
PD-L1: programmed death-ligand 1
PRAME: preferentially expressed antigen in melanoma
PSA: prostate-specific antigen
PSA50: PSA decrease >50% compared with baseline
PSMA: prostate-specific membrane antigen
RCC: renal cell carcinoma
ROR1: receptor tyrosine kinase-like orphan receptor 1
SCLC: small cell lung cancer
SSTR2: somatostatin receptor 2
STEAP1: six-transmembrane epithelial antigen of the prostate 1
TAA: tumor-associated antigen
TCE: T cell engager
TCER: T cell engaging receptors
TCR: T cell receptor
TILs: tumor-infiltrating lymphocytes
TME: tumor microenvironment
TMEFF2: transmembrane protein with EGF-like and two follistatin-like domains 2
TRAE: treatment-related adverse events
TRACTr: tumor activated T cell engager
TRAFsome: T-cell redirecting antibody fragment-anchored liposome
Treg: regulatory T cell
TYRP1: Tyrosinase-related protein 1.
