# Supplementary material for: Current landscape of T-cell engagers in early-phase clinical development in solid cancers
Source: Front Immunol. 2025 Oct 6;16:1665838. doi: 10.3389/fimmu.2025.1665838 (PMC12536262; doi:10.3389/fimmu.2025.1665838)
Supplement: Supplementary file 1 [file DataSheet1.docx]

Supplementary Material

# Supplementary Table 1. T-cell engagers approved by the U.S. Food and Drug Administration.

| **Agent** | **Tumor**  **antigen** | **Indication** | **Approval date** | **Approval type** | **Clinical trial(s)** |
| --- | --- | --- | --- | --- | --- |
| Tarlatamab | DLL3 | SCLC  (platinum resistant) | May 2024 | Accelerated | NCT05060016 |
| Elranatamab | BCMA | Multiple myeloma  (R/R) | August 2023 | Accelerated | NCT04649359 |
| Talquetamab | GPRC5D | Multiple myeloma  (R/R) | August 2023 | Accelerated | NCT03399799 |
| Glofitamab | CD20 | DLBCL  (R/R) | June 2023 | Accelerated | NCT03075696 |
| Epcoritamab | CD20 | DLBCL, HG-BCL  (R/R) | May 2023 | Accelerated | NCT03625037 |
| Mosunetuzumab | CD20 | Follicular lymphoma  (R/R) | December 2022 | Accelerated | NCT02500407 |
| Teclistamab | BCMA | Multiple myeloma  (R/R) | October 2022 | Accelerated | NCT03145181 NCT04557098 |
| Tebentafusp | gp100 | Uveal melanoma  (first-line) | January 2022 | Regular | NCT03070392 |
| Blinatumomab | CD19 | B-ALL  (consolidation) | June 2024 | Regular | NCT02003222 |
|  |  | B-ALL  (R/R) | July 2017 | Regular | NCT02013167 |

B-ALL: B-cell acute lymphoblastic leukemia; BCMA: B-cell maturation antigen; CD19: cluster of differentiation 19; CD20: cluster of differentiation 20; DLBCL: diffuse large B-cell lymphoma; DLL3: delta-like ligand 3; GPRC5D: G protein-coupled receptor class C group 5 member D; gp100: glycoprotein 100; HG-BCL: high-grade B-cell lymphoma; R/R: relapsed/refractory; SCLC: small-cell lung cancer.

## Supplementary Figures

**Maximum Tolerated Dose**


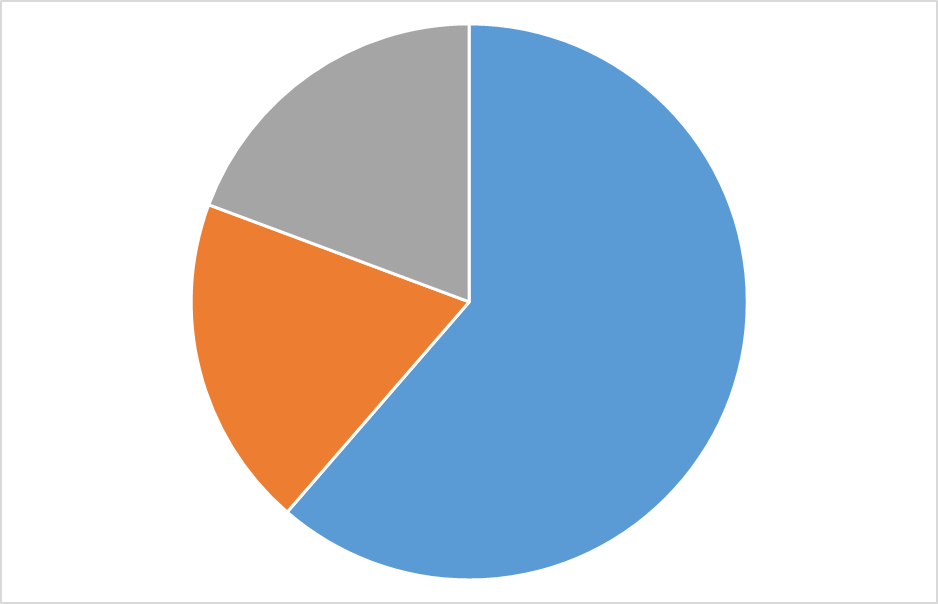


Not reached

61.3%

Reached

19.3%

Not reported

19.3%

| **Clinical trials** | | | |
| --- | --- | --- | --- |
| **Completed** | **Terminated** | **Ongoing** | **N/A** |
| **NCT02535078** | **NCT03577028** | **NCT05997615** | **NCT05543330** |
| **NCT04551352** | **NCT02659631** | **NCT05278832** | **NCT04128423** |
| **NCT04740034** | **NCT03515551** | **NCT05164458** |  |
| **NCT01723475** | **NCT05013554** | **NCT05359445** |  |
| **NCT02748837** |  | **NCT05958121** |  |
| **NCT02324257** |  | **NCT04429087** |  |
| **NCT03411915** |  | **NCT04471727** |  |
| **NCT04991740** |  | **NCT05387265** |  |
| **NCT03792841** |  | **NCT05356741** |  |
|  |  | **NCT03564340** |  |
|  |  | **NCT04221542** |  |
|  |  | **NCT04397276** |  |
|  |  | **NCT03448042** |  |
|  |  | **NCT04262466** |  |
|  |  | **NCT05450562** |  |
|  |  | **NCT04104607** |  |

**Supplementary Figure 1.**

Percentage of the early-phase clinical trials of T-cell engagers in solid tumors where the maximum tolerated dose could be determined or was not reached. For this analysis, we selected the dose-escalation studies whose clinical data have been disclosed between January 1, 2020, and April 30, 2025.
